# Dissociation of depression from apathy in traumatic brain injury: a case report

**DOI:** 10.1590/S1980-57642013DN70300014

**Published:** 2013

**Authors:** Raquel Quimas Molina da Costa, Fábio Henrique de Gobbi Porto, Rogério Paysano Marrocos

**Affiliations:** 1 MD. Universidade Federal do Estado do Rio de Janeiro (Unirio). Escola de Medicina e Cirurgia (EMC). Hospital Universitário Gaffrée e Guinle (HUGG). Departamento de Medicina Especializada. Disciplina de Psiquiatria. Serviço de Psiquiatria.; 2 MD. Behavioral and Cognitive Neurology Unit, Department of Neurology, and Cognitive Disorders Reference Center (CEREDIC). Hospital das Clínicas of the University of São Paulo, São Paulo, SP, Brazil.; 3 MD, MSc. Universidade Federal do Estado do Rio de Janeiro (Unirio). Hospital Universitário Gaffrée e Guinle (HUGG).

**Keywords:** apathy, depression, traumatic brain injury

## Abstract

Although not evident clinically, lesions to the prefrontal cortex cause great social and functional impairment to patients. The anterior cingulate cortex is intimately involved with motivational behavior and after injury to this area the onset of an apathetic state can be observed. This paper describes the case of a patient with traumatic brain injury to the prefrontal lobe presenting with a depressive syndrome associated with apathetic symptoms. After appropriate treatment for depression, intense apathy was revealed, an irreversible sequelae of the traumatic brain injury, constituting the main barrier to the patient's return of lifestyle and independence.

## Abstract (pt)

Apesar de não se destacarem ao olhar clínico, as lesões do córtex pré-frontal acarretam grande prejuízo funcional e social aos pacientes. O córtex do cíngulo anterior está intimamente envolvido com o comportamento motivacional e o que se observa após um dano a esta região é a instalação de um estado apático. Este trabalho visa relatar o caso de um paciente com lesão traumática do lobo pré-frontal que apresentou uma síndrome depressiva associada a sintomas apáticos. Após tratamento adequado da depressão foi possível perceber intensa apatia, sequela irreversível do traumatismo crânio encefálico, que se constituiu como principal obstáculo ao retorno do estilo de vida e independência do paciente.

## INTRODUCTION

Lesions of the prefrontal cortex are often not easily recognized without a thorough neurological, cognitive and neuropsychiatric examination. Frequently, more evident elementary neurologic deficit may mask the behavioral symptoms. Although not evident in a casual encounter, lesions of the prefrontal cortex can cause great social and functional impairment in affected subjects, as is often reported by them and their relatives.^1^

The prefrontal cortex can be subdivided into three major areas, each of which is predominantly related to distinct cognitive functions. The orbitofrontal cortex (OFC) is associated with the regulation of social behaviors and social cognition. Damage to its structures is associated with changes in personality, resulting primarily in disinhibition and disregard for social rules.^1^ The dorsolateral cortex (DLC) is the region related to executive functions such as task organization, planning, hypothesis generation and decision making while the anterior cingulate cortex (ACC) is intimately involved with motivational behavior where damage to this region may result in lack of initiative, motivation and volition, with the onset of an apathetic state.^1^

Apathy has been defined in different ways in the literature, but lack of motivation can be identified as the main feature in most of these definitions.^2^ Motivation is understood as the direction, intensity and persistence of goal-directed behaviors.^3^ Apathy may have several dimensions, such as motor apathy, causing akinetic mutism, cognitive apathy, decreasing the curiosity and interest in learning, affective apathy, with reduced facial expression, emotional apathy, with reduced social interest and affection, and motivational apathy causing decreased initiative. Lesions in the ACC impact motivational brain processing, the linking of external stimuli to the needs of the internal milieu.^4^ Bilateral damage to the ACC causes akinetic mutism, in which patients are deeply impaired, rarely move and eat only when fed by others, the so-called apathetic abulic state.^5^ Unilateral injury causes less severe apathetic abulic syndrome, but may present as transient akinetic mutism.^5^

Mood changes are also frequently seen in cases with prefrontal lesions^6^ and major depression is considered a common sequela in traumatic brain injury. Depression is associated with worse functional and cognitive recovery.^7,8^

Herein, we reported a case of a patient with traumatic injury to the prefrontal cortex, causing a dysexecutive syndrome, depressive symptoms and apathy. In this case, apathy was only evidenced by a thorough neuropsychiatric and cognitive evaluation and the application of dedicated scales. We emphasize the differences between apathy and depression, review the neuroanatomy of regions linked to apathy and discuss the management of apathetic patients.

## CASE REPORT

A 37-year-old right-handed man, without previous psychiatric history was evaluated in the outpatient neuropsychiatric unit. His history began eight years prior when he was struck by a train, resulting in a traumatic brain injury and multiple skull fractures. He was diagnosed with left-side epidural hematoma and submitted to frontotemporal craniotomy for drainage. He remained in a coma for 32 days, slowly recovering his level of consciousness at the time.

In the first interview, eight years after the accident, he showed depressed mood and spoke little. He cried easily and reported unsatisfactory sleep. His wife, who only met him after the accident, said that he did not like to go out, had no interest in friends, ate only when someone prepared his food for him and could go all day long without eating. He watched television but was only interested in soccer matches. He had little initiative, spent many hours lying down "thinking" (as he described) and had no interest in sex. This pattern of behavior intermittently changed to outbursts of aggression, stubbornness and irritability. The wife stated, however, that he used to be affectionate with her and their daughter.

**Figure 1:**
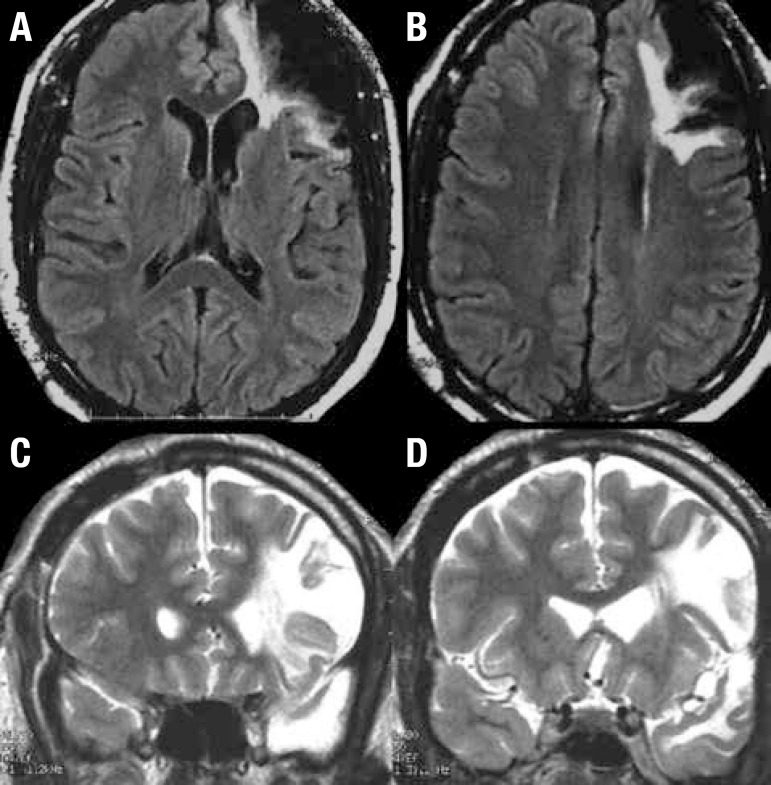
Brain MRI. [A, B] Axial FLAIR. [C, D] coronal T2WI. Reveals a large area of encephalomalacia and surrounding gliosis involving left frontal lobe. There is ex-vacuo dilatation of the left lateral ventricle.

In the following appointment, his brother reported that before the accident he was "playful", had many friends, liked to hang out with them and was very cheerful. He loved playing football, was very affectionate and described by others as "a sweet person". He noted that after the accident he had become cold emotionally, fat, lacked energy, was very aggressive and could not control his emotions, once even threatening his father. During the examination he was lucid and oriented, his language abilities were preserved and his general neurological exam was normal. Magnetic resonance image showed extensive injury to the left prefrontal DLC and ACC (Figure 1).

He was submitted to neurocognitive and functional evaluation and his performance is shown in Table 1.^9-15^ Performance on the Stroop Test (ST), Trail Making Test (TMT) and Wisconsin Card Sorting Test (WCST) was abnormal. He was treated for the depressive syndrome with nortriptyline 25 mg, twice a day. After 10 weeks of treatment for depression, he reported feeling better regarding sadness, had stopped crying and was sleeping well. He still had no interest in sex and only ate when his wife prepared his meal. His wife said that he did not finish daily tasks which he had started and could not do two things at the same time. She also said that he was less aggressive, but kept lying still for many hours, "without doing anything". When asked about it, he didn't show any critical judgment of his current condition. After 2 months of treatment, his wife was able to find him a job in a local bus company. She reported that she needed to accompany him to work every day and this was the only way he was able to keep his job. He is still being followed and has remained stable ever since.

**Table 1:** Patient performance before and after antidepressant treatment.

	Performance in first visit	Performance in follow-up visit(after antidepressant treatment)	Cut-off	Range
FAB	10	11	12	0-18
MADRS	24	9	Mild: 7-19 Moderate: 20-34 Severe: >34	0-60
MMSE	27	29	24*	0-30
AS	33	37	13\14	0-42
GAF	50	50	80	0-100
SOFAS	45	45	80	0-100

AS: Apathy scale9; FAB: Frontal assessment battery^10^; GAF: Global Assessment of Functioning^11^; MADRS: Montgomery and Asberg depression rating scale^12^; MMSE: Mini mental statement examination^13^,^14^; SOFAS: Social and Occupational Functioning Assessment Scale1^5^.

* According to educational level.

## DISCUSSION

This case report shows the consequences of an injury to the prefrontal cortex causing apathy and depression simultaneously. Although similar, the two entities are not the same.

Major depression is characterized by depressed mood, loss of interest or pleasure in nearly all activities and symptoms such as sleep disturbances, appetite changes, decreased psychomotor activity, decreased energy, difficulty concentrating and thoughts of worthlessness or guilt.^7,16^ Some studies in the 1990s correlated a higher incidence of depressive symptoms with lesions in the left frontal pole and in some cases, a significant correlation between the severity of the symptoms and the distance from the anterior edge of the cerebral lesion to the frontal lobe.^1,8^

Apathy is more closely linked to the "lack of motivation" concept.^2^ Motivation is understood as the direction, intensity and persistence of goal-directed behaviors.^3^ It can be evidenced by a reduction of directed behavior, lack of energy, effort and need for external orders to carry tasks out. Concomitantly, changes in goal-direct cognition, for example, lack of interest in learning new things and loss of concern about personal problems also often occur. Behavioral abnormalities such as lack of emotional reaction (both positive and negative) and the flat affect also occurs. Apathy is a clinical manifestation that can be measured by some dedicated scales such as the Apathy Scale proposed by Stekenstein and validated in Portuguese.^2,5,9^

The symptomatology related to apathy can be misdiagnosed as depression. Sometimes, depression may cause some apathetic symptoms, but the two conditions are nosologically distinct. The importance of separating these syndromes hinges on the fact that depression and apathy have different types of management and prognosis. Depression has a better response to treatment, as seen in this patient. In fact, once treated, the mood disorder presented by the patient improved yet the apathetic symptoms became more evident and remained chronic. In this case, apathy was the main complaint reported by his family and his biggest obstacle to returning to an independent and working life.

In this patient, there was also great impairment in executive functions due to the extensive lesion to the DLC. A dysexecutive syndrome was noted through his low performance on the WCST, FAB, ST and TMT. During the interview, specific complaints were identified, such as "he does not finish the tasks he started", "cannot do two things at the same time", which are characteristic difficulties of dysexecutive syndrome.^17^

Due to the extensive prefrontal lesion, there were also behavior changes, such as aggressiveness and explosive temper, which are typically related to the OFC. As traumatic brain injuries are often not selective to any prefrontal region, widespread damage usually occurs, generating a "blend" of frontal symptoms. Furthermore, it is important to remember the presence of brain circuits between the prefrontal cortices, which further complicates the occurrence of a clinical presentation with symptoms unique to one or another region.^1^

A Japanese study published in 2011 evaluated depressive and apathetic symptoms in patients with traumatic brain injury and correlated them with the Functional Independence Measurement. The authors found that apathy was more strongly associated with a negative impact on the recovery of these patients than depressive symptoms.^7^ This patient has a lesion in the left anterior pole of the frontal lobe, a site often related to the onset of depressive symptoms compared with lesions in other areas or even in the right frontal pole.^18,19^

These patients find it very difficult to keep or even get a new job, because of the dysexecutive syndrome, lack of motivation and interest and also the difficulty in relating normally with colleagues.^20^ Sometimes this is only possible with the continued effort of the family, as seen in this case. The great social impairment and functional disability of the injuries involved are evident through relatively low GAF and SOFAS.

The appropriate investigation of cognitive impairment and patient behavior is the first step to identify their limitations and guide a possible rehabilitation. Identifying these deficits is important for the family and the patient to understand these changes and know what to expect in the future. It is essential to provide adequate treatment for those manifestations that may show improvement with drug intervention and to identify possible comorbid psychiatric disorders in order to take the necessary therapeutic approach. Often the damage is perceived months or years after the trauma but can cause long-term disability and major functional impairment.^21^

However, it is curious to note that these changes were not obvious or readily related to the trauma, as would be the case in a motor, aphasic or visual-perceptual lesion. The sequelae related to prefrontal damage are difficult to measure, often requiring several questionnaires, scales and tests to be able to draw a parallel between the anatomical lesion and functional impairment and consequently allow devising of a treatment and/or rehabilitation plan tailored to these. The introduction of drug therapy in this case exemplifies that depressive symptoms are potentially treatable and therefore reversible. The same is not true however for apathetic symptoms.

## Figures and Tables

**Table 2:** Patient performance on executive function tests:

Test	Patient	Normative values
DSF	4	6 to 7
DSB	3	4 to 5
TMT A	37"	(mean=35.8; SD =11.9)
TMT B	5'43''	(mean=81.2; SD = 38.5)
ST 1	19.9'	(mean=12.56; SD=1.89)
ST 2	18.6'	(mean=16.16; SD=3.46)
ST 3	21.3'	(mean=31.32; SD=8.22)

ST: Stroop test; TMT: Trail Making test; DSF: Digit Span Forward; DSB:Digit Span Backward.
